# Decoupling Design from Darwinian and Skinnerian Selection

**DOI:** 10.1007/s40614-025-00476-y

**Published:** 2025-09-15

**Authors:** Edward A. Wasserman

**Affiliations:** University of Iowa, Iowa City, IA United States

**Keywords:** Evolution, Reinforcement, Variation, Selection, Retention

## Abstract

Darwin explained how seemingly purposeful and foresightful design could be decoupled from natural selection. Skinner followed suit for selection by reinforcement. Together with cultural selection, these three selectionist ideas represent today’s prime pillars of evolutionary thinking in biological and behavioral science. Nevertheless, controversy continues because mentalism still creeps into the efforts of psychologists, biologists, and philosophers to meet the interpretive challenges posed by creative behaviors and innovations. A prime problem may be the unfortunate connotations accompanying the term “selection.”

[I]f mindless evolution could account for the breathtakingly clever artifacts of the biosphere, how could the products of our own minds be exempt from an evolutionary explanation? (Dennett, 1995, p. 37)

Natural selection transforms ancestral contingencies into the appearance of design; selection by reinforcement transforms individual contingencies into the appearance of intention. (Donahoe, 2003, p. 111)

Theologian and philosopher William Paley (1743–1805) memorably likened design in the natural world to design in the realm of human manufacture (Gregory, 2009b). In his *Natural Theology* (1802/2009), Paley believed both the eye and the telescope to have been intelligently designed. For him, their evident complexity and functionality required each having been created by an intelligent designer: an omniscient deity for the eye and a prospicient human for the telescope. Of these two objects, Paley wrote:

As far as the examination of the instrument goes, there is precisely the same proof that the eye was made for vision, as there is that the telescope was made for assisting it. They are made upon the same principles; both being adjusted to the laws by which the transmission and refraction of rays of light are regulated. (pp. 19–20)

Biologist Charles Darwin (1809–1882) pondered the provenance of two different objects: the hinge of a bivalve mollusk and the hinge of a door. He proposed that the mollusk’s hinge needed no designer at all: operating over a prolonged period of time, natural selection alone could effectively produce this exquisitely articulated structure (Crane & Denny, 2023). In this case, Darwin contended that: “The old argument of design in nature, as given by Paley, which formerly seemed to me so conclusive, fails, now that the law of natural selection has been discovered.” He then speculated that: “We can no longer argue that . . . the beautiful hinge of a bivalve shell must have been made by an intelligent being, like the hinge of a door by man” (Darwin, 1887/1958, p. 87).

Darwin’s revolutionary selectionist theory famously focused on the evolution of nature’s products—with sweeping and possibly dangerous repercussions (Dennett, 1995). Yet, by implying that the door hinge may have required the participation of an intelligent human designer, Darwin seems to have accepted that a stark contrast holds between the evolution of nature’s products and the evolution of humans’ products.

If this were all one were to know about Darwin’s theorizing, then he might unnervingly be judged to have been a naturalist when dealing with the origin of species, but a mentalist when dealing with the origin of human actions and artifacts (see Wasserman & Blumberg, 2010, for further discussion of this matter). Yet, Darwin’s other writings help clarify this vexing paradox; they concerned humans’ selective breeding of plants and animals. This breeding practice—inaccurately dubbed artificial selection for the simple reason that we ourselves are part of nature (Burnett, 2009)—was to play a prime part in Darwin’s developing theory (Ayala, 2007; Costa, 2009; Gregory, 2009a, b; Theunissen, 2012). In the words of Wilner (2006): “Darwin’s theorizing was a spiraling speculation that was both fed and constrained by the results of the breeders’ intervention in nature” (p. 32).

Darwin’s studies of selective breeding will serve as the impetus for my upcoming discussions concerning: the participation of natural selection in the origin of species, the role of reinforcement in promoting adaptive behavior in individual organisms, and the impact of cultural selection in advancing human social practices. The ongoing debate between mentalism and behaviorism will figure prominently in all of these discussions.

## Darwin and Selective Breeding Practices

In a letter to Asa Gray 2 years prior to publishing *On the Origin of Species* (1859), Darwin disclosed that,

. . . all my notions about *how* species change are derived from long-continued study of the works of (& converse with) agriculturists & horticulturists; & I believe I see my way pretty clearly on the *means* used by nature to change her species & adapt them to the wondrous & exquisitely beautiful contingencies to which every living being is exposed. (quoted by Gregory, 2009a, p. 6)

Indeed, the same year in which he published the *Origin*, Darwin wrote to Alfred Russell Wallace that: “I came to the conclusion that selection was the principle of change from the study of domesticated productions; and then, reading Malthus, I saw at once how to apply this principle” (quoted by Gregory, 2009a, p. 6).

Darwin gleaned even more from his assiduous study of selective breeding than his insights into the law of *natural selection*; he further suggested that *artificial selection* need not always have been practiced with meticulous rigor or with a clear goal in mind. Rather, Darwin (1959) hypothesized that breeders might also have engaged in the possibly *more* significant practice of what he called “unconscious” or unpremeditated selection (Burnett, 2009; Gregory, 2009a):

At the present time, eminent breeders try by methodical selection, with a distinct object in view, to make a new strain or sub-breed, superior to anything existing in the country. But, . . . a kind of Selection, which may be called Unconscious, and which results from every one trying to possess and breed from the best individual animals, is more important. Thus, a man who intends keeping pointers naturally tries to get as good dogs as he can, and afterwards breeds from his own best dogs, but he has no wish or expectation of permanently altering the breed. Nevertheless I cannot doubt that this process, continued during centuries, would improve and modify any breed. (p. 4)

Darwin’s most clearcut and convincing example of unconscious selection involved an exotic breed of pigeon—the fantail (Secord, 1981). Today, as in Darwin’s time, pigeon fanciers might proudly trumpet the success of their efforts to maintain or to improve their stock of fantails by deploying what many would call conscious and premeditated selective breeding. Yet, Darwin (1859) went to special lengths to disabuse his readers of any such deliberate efforts in the *creation* of the fancy fantail:

To use such an expression as trying to make a fantail, is, I have no doubt, in most cases, utterly incorrect. The man who first selected a pigeon with a slightly larger tail, never dreamed what the descendants of that pigeon would become through long-continued, partly unconscious and partly methodical selection. (p. 39)

In Darwin’s estimation, intelligent *human* design was not involved in the origin of the fantail. Nor, in an analogous way, was intelligent *divine* design involved in the origin of the rock dove—the very breeding stock from which fantails were to be derived.

Of course, Darwin’s initial conjectures about selective breeding are exceedingly difficult to confirm. As was true for the origin of species, Darwin was acutely aware of how and why so little was known about the origin of domestic breeds and the possible breeding techniques used to produce them. He particularly appreciated that inherited variations might change only subtly from generation to generation over a great many years:

[A] breed, like a dialect of a language, can hardly be said to have had a definite origin. A man preserves and breeds from an individual with some slight deviation of structure, or takes more care than usual in matching his best animals and thus improves them, and the improved individuals slowly spread in the immediate neighbourhood. But the chance will be infinitely small of any record having been preserved of such slow, varying, and insensible changes. (Darwin, 1859, p. 43)

Darwin was thus entirely open to unconscious and unplanned human selection participating in the provenance of plant and animal varieties. Yet, he appears not to have seriously engaged the closely related and extremely important possibility of such impromptu selection participating in the origin of innumerable other products of human behavior, such as a door hinge, a telescope (Simonton, 2012), or the law of natural selection (Costa, 2009). The possible involvement of unconscious and unpremeditated selection in human conduct was later to become a prominent concern of behavioral psychologist B. F. Skinner (1904–1990).

## Skinner and Selective Reinforcement Processes

In this effort, Skinner can be seen as dramatically advancing the early speculations of Edward L. Thorndike (1874–1949) concerning the mechanisms of learning in individual organisms. Thorndike (1898) taught cats to press a bar or to slide a latch by regularly following these responses with escape from a small puzzle box and a tasty tidbit of fish. Thorndike believed such release and food reinforcement to have stamped in the association between entry into the box and the required response. The general decline in his cats’ time to escape and obtain food convinced Thorndike that associative learning was not a sudden insightful process; rather, it was an altogether blind, unconscious, and automatic hit-or-miss affair.

This was a critical point, because for neither Thorndike nor Skinner was the behaving organism properly deemed to be the intelligent selecting agent for the development of learned actions. Instead, the environmental contingency between responses and reinforcers was responsible for promoting adaptive behavior by means of a second selectionist process (Dennett, 1975; Donahoe, 2003): What Thorndike called the *law of effect* and Skinner called *selection by reinforcement* automatically increased the likelihood of successful acts and decreased the likelihood of unsuccessful ones.

Skinner therefore regarded Darwin as having replaced a purposeful intelligent deity with a natural selective process to produce changes in human anatomy and physiology: “Evolutionary theory moved the purpose which seemed to be displayed by the human genetic endowment from antecedent design to subsequent selection by contingencies of survival” (Skinner, 1974, p. 224). Skinner’s own operant conditioning theory involved a related conceptual replacement, in which the purposeful intelligent mind was replaced by a different selective process: “Operant theory moved the purpose which seemed to be displayed by human action from antecedent intention or plan to subsequent selection by contingencies of reinforcement” (p. 224).

At this juncture, we can see that the law of natural selection and the law of effect face a profound and common challenge: namely, the origin of novelty. Appreciating this challenge, Skinner (1974) noted the parallel between the contingencies of reinforcement and the contingencies of survival: “Natural selection explained the origination of millions of different species, without appealing to a creative mind” (p. 224). In the case of human behavior: “contingencies of reinforcement may explain a work of art or a solution to a problem in mathematics or science without appealing to a different kind of creative mind or to a trait of creativity” (p. 224). Thus, Skinner argued that, “As accidental traits, arising from mutations, are selected by their contribution to survival, so accidental variations in behavior are selected by their reinforcing consequences” (p. 114).

Skinner’s extension of Darwin’s evolutionary theory to novel human action was bold and controversial. Indeed, Dennett (1995) considered this specific extension to be tantamount to quashing “the illusion of human authorship, our own divine spark of creativity and understanding” (p. 37). The shock of such a dramatic disavowal, Dennett anticipated, would be bound to evoke the overpowering fear that evolutionary theory “will not just explain but will explain *away* the minds and purposes and meanings that everyone holds dear” (p. 40).

Contending with that fear is just one part of the challenge facing science today; the other, is to ascertain just how apt the extension truly is. How might evolutionary theory be effectively applied to human authorship, purpose, design, and creativity?

## Evolutionary Theory, Design, and Creativity

Particularly problematic for progress in this realm of inquiry has been the concept of creativity—commonly defined as a venerable psychological trait, enjoyed at the highest level by a select few. This baldly elitist sense of creativity has been duly criticized (Baer, 2022; Shapin, 2020) as has the lack of integration of creativity into evolutionary and neuroscientific thinking (Dietrich & Haider, 2015). Nonetheless, efforts to deal directly with creative behaviors and products suggest promising areas of future investigation. Let me now discuss a few of them (also see Goddard, 2021, and Morgan et al., 1992, for other lines of inquiry).

One significant and biologically inspired line of exploration concerns the sound and shape of the violin—a musical instrument that has markedly evolved as a result of the diverse construction methods of its renowned makers. Nia et al. (2015) studied the geometry of 470 violins’ twin sound-holes (or f-holes). This team reported that these curvaceous slits gradually changed over several centuries “from simple circular openings of tenth century medieval [fiddles] to complex f-holes that characterize classical seventeenth-eighteenth century Cremonese violins” (p. 2). These physical modifications measurably altered the acoustic power of the instrument. It is critical that these changes did not occur quickly, as might have been the case if they had sprung from a sudden, insightful, or preconceived change in violin design.

Utilizing concepts and equations from evolutionary biology that measure generational fluctuations in gene frequency, Nia et al. (2015) proposed that “the gradual nature of the sound hole changes from the tenth to sixteenth century . . . is consistent with incremental *mutation* from generation to generation of instruments” (p. 8, emphasis added). The authors further speculated that these structural changes were likely to have emerged *by chance* and were acted on by “a *selection* process favoring instruments with higher power efficiency” (p. 8, emphasis added), consistent “with evolution via *accidental* replication fluctuations from craftsmanship limitations and subsequent selection” (p. 11, emphasis added).

Along with these demonstrably *functional* changes in violin fabrication were entirely *aesthetic* changes. Chitwood (2014) studied the overall shape of Cremonese violins—a physical attribute that experts do not believe significantly affects sound power and quality, unlike changes in sound-hole geometry. Chitwood’s biostatistical or “morphometric” analysis pointed to a striking parallel between the factors leading to changes in the shape of the violin over hundreds of years of human construction and changes in the shape of plants and animals over millions of years of biological evolution.

Chitwood found that most of the violins from famed luthiers clustered in just four groups, indicating that “during long years of apprenticeship within a workshop . . . peculiarities in the design and shape of instruments, transmitted luthier-to-apprentice, would arise, not unlike *genetic drift”* (p. 9, emphasis added). These biological parallels suggested to Chitwood a plausible account of diverging lineages in the shape of violins that involved random variations in a common ancestor subsequently becoming fixed within individual familial lines. In this case, these changes were not due to *function*, but to *fancy*—in much the same way as Darwin had analyzed the creation of numerous exotic pigeon varieties resulting from the selective breeding practices of human pigeon fanciers (Gregory, 2009a; Secord, 1981).

As a plant biologist, Chitwood (2014) found it unsurprising that the inheritance of violin morphology could be affected by analogous natural evolutionary processes, because the instrument was “crafted by *living organisms, themselves subject to natural laws*” (p. 11, emphasis added). However, neither he nor Makris were familiar with the law of effect (personal communications, October 2015). Nevertheless, behavioral science can credibly explain how the law of effect could have influenced the evolution of the violin (Wasserman & Cullen, 2016).

In the case of sound-hole geometry, progressive increases in length yielded increases in the violin’s acoustic power (Nia et al., 2015). This rise was to become increasingly important as larger concert venues came to require greater audible volume (Nia et al., 2015), thus increasing the appeal of these instruments to prospective buyers. Although changes in violin shape were unlikely to have produced audible effects, they too may have been preferably chosen because of customers’ demand for different styles (Chitwood, 2014). It should be noted that each these evolutionary changes appears to have arisen in especially small and virtually imperceptible steps, consistent with the idea that the violin’s evolution involved a prolonged hit-or-miss, trial-and-error process in which the violin’s early makers could have had no preconception of its now-revered form and construction details. Thus, variation and selection are more likely to have produced the modern violin than premeditation.

A far less detailed historical method has been deployed to explore the genealogy of an idea, behavior, or product across different people over multiple lifetimes. For instance, Brandt and Eagleman (2017) traced the lineages of such diverse creations as Henry Ford’s automobile assembly line, Samuel Taylor Coleridge’s epic poem *Rime of the Ancient Mariner*, and Pablo Picasso’s pioneering cubist portrait *Les Demoiselles d’Avignon*. Brandt and Eagleman conclude that creative ideas are rarely altogether new; instead, they evolve from earlier memories and impressions, often involving bending, blending, and breaking. In bending, an original idea is altered or twisted from its normal shape. In blending, two or more ideas are merged. And, in breaking, whole ideas are disassembled and their pieces are reassembled into a new entity.

Conniff (2008) arrived at a similar conclusion concerning generational progression. Discussing the evolution of the theory of evolution by natural selection, Conniff observed that:

We call it Darwinism, for short. But in truth, it didn’t start with Darwin, or with Wallace either, for that matter. Great ideas seldom arise in the romantic way we like to imagine—the bolt from the blue, the lone genius running through the streets crying, “Eureka!” Like evolution itself, science more often advances by small steps, with different lines converging on the same solution.

A complementary approach explores the genealogy of an idea, behavior, or product within a single person’s lifetime, relying on first-hand accounts of the innovators themselves if available. Wasserman (2021) applied this tactic to such varied innovations as Dick Fosbury’s “flopping” style of high jumping, Ignacio Ponseti’s nonsurgical treatment of clubfoot, and Ansel Adams’s fine art techniques of black-and-white photography. These and countless other innovations can be seen to result from the confluence of three factors: the current context or *Zeitgeist,* coincidental or chance occurrences*,* and the consequences of the innovation itself.

Among the most resourceful of such genealogical endeavors is Simonton’s (2007) exploration of Picasso’s preliminary sketches of the figures that would ultimately appear in his intensely emotional oil painting *Guernica*. At issue was whether Picasso’s sketches implied a series of expert monotonic improvements or merely a series of blind nonmonotonic variants. Simonton concluded that Picasso, “was largely accumulating several possible variants of each main figure and only later selected the final representation from that set. But because creativity relies on a process of blind variation, he could not see where he was going until after he got there” (p. 340). (Further discussion of the role of ‘blindness’ in the context of behavioral variation and selection can be found in Dietrich & Haider, 2015, and Simonton, 2023.)

A similar conclusion holds for the evolution of Post-It notes (Wasserman, 2023), which involved two contemporaneous 3M employees: Spencer Silver, who accidentally concocted a weak and seemingly useless adhesive, and Art Fry, who, through extensive trial-and-error experimentation, eventually stumbled on the idea of applying that adhesive to small squares of paper to serve as detachable notes. At no point along their meandering 12-year journey did either Silver or Fry have any inkling what the final wildly popular office product would be.

Even more probing archaeological research has used powerful x-ray fluorescence to examine the 3,000-year-old paintings inside Egypt’s Theban Necropolis (Martinez et al., 2023). That novel technology allows one to “see” any layers of paint that may lie beneath the surface of this extraordinary wall art. Being familiar with the appearance of the deceased persons ought to have provided the painters with a clear visual memory to guide their work. Nevertheless, several layers of paint were surprisingly revealed using this technological procedure, thereby suggesting that the artisans may have engaged in an iterative process involving several revisions. Philippe Martinez, the study’s lead author, made an interesting observation: “The fact that they altered paintings at all challenges many historians’ assumptions about Egyptian art as preplanned and precise. What we see is that nothing is perfect. And that’s great, because they were human beings” (Gupta, 2023).

Yet, the origin of *human* creativity and innovation greatly predates ancient Egypt. As told by archaeologist Chip Colwell (2023), the story goes back at least 3 million years to our *hominid* ancestors, for whom the participation of language would not even play a part. Knapping stone tools set in motion a series of profound evolutionary changes that prompted dramatic behavioral and biological changes that, through three extended phases, ultimately afforded us our current ability to create more “stuff” than we ever imagined or needed.

### Provisional Summary

Let me summarize the discussion so far. I began this article by introducing Paley’s argument from design—the notion that both nature’s creations and those creations of our own making result from foresighted, intelligent design. I next discussed how Darwin’s law of natural selection could explain the complexity and functionality of nature’s diverse life forms without the involvement of a divine designer.

I next considered Darwin’s further speculation that blind, unconscious selection might critically have participated in humans’ initial efforts in breeding plants and animals. As noted by Gregory (2009a): “unconscious selection involves no goal-directed choices. In this regard . . . there is no fundamental difference between unconscious selection and natural selection” (p. 23).

I then extrapolated Darwin’s evolutionary analysis to other realms of human endeavor and raised the possibility that a second selectionist law—one also decoupled from premeditated design—might participate in our own creation of novel ideas, behaviors, and devices. In particular, the law of effect, introduced by Thorndike and further developed by Skinner, suggests that the many things we do and make—including styles of Olympic high jumping, medical cures, narrative poems, works of visual art, scientific theories, violins, and office paper products—may have had their origins in an analogous, hit-or-miss process.

But what about Darwin’s distinction between unconscious and methodical selection in horticulture and animal husbandry? How might that distinction apply to other realms of human behavior? Let’s revisit Darwin’s example of the hinge of a mollusk and the hinge of a door for a valuable clue.

Darwin was certainly not alone in seeing a salient disparity between nature’s creations and humans’ creations. Other theorists too have questioned any correspondence between their origins. Consider the perspective of François Jacob (1977). He judged as unsuitable any resemblance between the process of organic evolution and the modus operandi of a human engineer (also see Ayala, 2007; Dawkins, 1987):

[I]n contrast to what occurs in evolution, the engineer works according to a preconceived plan in that he foresees the product of his efforts. . . . However, if one wanted to play with a [possibly suitable] comparison, one would have to say that natural selection . . . works like a tinkerer . . . who does not know exactly what he is going to produce but uses whatever he finds around him . . . to produce some kind of workable object. . . . What he ultimately produces is generally related to no special project, and it results from a series of contingent events. (Jacob, 1977, pp. 1163–1164)

A similar contention was made by Doebley (2006) in specific reference to Darwin’s discussion of the parallel between natural selection and unconscious selective breeding: “Tinkering . . . is the order of the day in domestication as in natural selection and Darwin’s use of domestication as a proxy for evolution under natural selection was, not surprisingly, right on the mark” (p. 1319).

## Cultural Selection

Perhaps we might be able to reconcile these decidedly different views of human innovators as tinkerers or engineers by appealing to a third evolutionary process: namely, *cultural selection*. Put simply, yesterday’s tinkerers may have gradually become today’s engineers, thanks initially to oral traditions and subsequently to written words, detailed diagrams, and prototypes (Kubina et al., 2006; Stahlman & Leising, 2018)—all representing the cumulative result of the law of effect operating across generations of individuals.

Indeed, Skinner (1981) explicitly invoked the advent of verbal behavior as effectively propelling people’s transformative shift from tinkerers to engineers through a third kind of selection: namely, the evolution of social environments or cultures. (Subsequent to this, Baum [e.g., 2000] and Glenn [e.g., 2003] have more extensively and assiduously considered the participation of selectionism in cultural evolution). According to Skinner (1981):

The process presumably begins at the level of the individual. A better way of making a tool, growing food, or teaching a child is reinforced by its consequence—the tool, the food, or a useful helper, respectively. A culture evolves when practices originating in this way contribute to the success of the practicing group in solving its problems. It is the effect on the group, not the reinforcing consequences for individual members, which is responsible for the evolution of the culture. (p. 502)

In this way, Skinner envisioned a single overarching process—what he called *selection by consequences*—to embrace *phylogenic* changes in organisms’ structure and function unfolding across countless generations, *ontogenic* changes in acquired behaviors within an individual organism’s lifetime, and *sociogenic* changes in cultural practices, as learned behaviors are progressively passed from one individual to another (Stahlman & Catania, 2020).

Nonetheless, although it may be ego-inflating to luxuriate in the innumerable triumphs of today’s electrical, mechanical, civil, and genetic engineers, we should never complacently presume that unqualified success is the inexorable result of sociogenetic selection. As one vivid example, the remarkable feats of engineering that went into the construction and operation of the Deepwater Horizon later demanded desperate remedial efforts to quell the unremitting gushing of oil that was released after its unexpected and deadly explosion in 2010.

No matter how determined we may be to design the end of failure, this admirable goal seems to be inevitably and infuriatingly unattainable. It’s thus essential for us always to remember the humbling catchphrase of the late historian of engineering, Henry Petroski (2006, 2012): form doesn’t follow *function*, it follows *failure*. Even today’s best engineers must remain open to tinkering. There’s no escaping the law of effect.

## A Recent Resurrection of Paley’s Proposal and Darwin’s Design Paradox

The issues considered in the preceding paragraphs are among the most profound and contested of the past 2 centuries. It is remarkable that, in a pair of brief *New York Times* articles published within a week of one another (Johnson, 2014a, b), science journalist George Johnson not only pointedly engages many of those same issues, but in the process, he notes a startling theoretical near miss. Most important, he brings us full circle to Darwin’s design paradox.

Johnson begins his narrative with Paley’s (2009) famous inference that all objects of intricate construction, like watches, require a creator—in this case, a watchmaker. Of course, living beings too are intricately constructed. So, Paley inferred that they, and we, also had a creator. As God’s only rational handiwork, Paley believed that we humans would need convincing evidence of our own divine design. Paley thus proposed that it was because of the extraordinary complexity of our contrivance that the existence, agency, and wisdom of God could be revealed to us.

Johnson (2014b) next proceeds to restate Paley’s final point in more modern, comprehensible terms: “To appreciate God . . . we needed to sense that we were constructed in the same way we would make a robot.” Then, Johnson shrewdly turns the tables on Paley: “He didn’t consider that he might have things backward: that our image of God the machinist is a reflection of ourselves.” Touché!

But Johnson doesn’t seek to disparage Paley’s ideas. On the contrary, he notes how meticulous Paley’s observations of nature were and how tantalizingly close he came to espousing a modern evolutionary theory, one which presaged Darwin’s (1802) own process of natural selection:

The eye, the animal to which it belongs, every other animal, every plant, indeed every organized body which we see, are only so many out of the possible varieties and combinations of being, which the lapse of infinite ages has brought into existence; that the present world is the relict of that variety; millions of other bodily forms and other species having perished, being by the defect of their constitution incapable of preservation, or of continuance by generation. (p. 68)

Nevertheless, Johnson (also see Gregory, 2009b) appreciates that Paley never took the next steps to explaining “how something as intricate as life could emerge through trial and error” (Johnson, 2014b). Nor could Paley envision how “the brains and hands that design civilization’s artifacts are products of the same evolutionary algorithm—random generation and testing.”

Johnson (2014b) ultimately segues to Darwin and the theory of natural selection, noting that: “Half a century later came Darwin’s world-changing insight. While clocks and other machinery result from human craft, biological complexity blindly emerged from the tinkering and testing of evolution.”

So, just what did Johnson have to say about the creative behaviors of the watchmaker and the machinist? Do they painstakingly ply their craft according to a preconceived design and with a clear end in mind? Do they do so without the involvement of trial and error? Without any tinkering or testing? Might it not instead be the case that the very artifacts these workers produce with their brains and hands result from another closely related selectionist algorithm involving random generation and testing? Yes, I’m proposing the active participation of selection by reinforcement! It is sad that, much like Darwin, Johnson said nothing about this vital issue other than that these individuals use their brains and hands to design civilization’s artifacts.

### Dawkins’s Conjectures

Perhaps we might consult a different author who espouses a more fully developed understanding of design in evolution and behavior. Let’s turn to Richard Dawkins, the most prominent contemporary evolutionary biologist and author of the widely acclaimed book, *The Blind Watchmaker*—a book whose title gives away its principal thesis.

As did Paley, Dawkins also contemplates possible parallels between the telescope and the eye as well as between the watch and a living organism—all extraordinarily complex entities. Dawkins senses no mystery as to how the telescope and the watch came into existence; they were made by humans with foresighted purpose in their mind’s eye. For Dawkins, the real mystery to be solved is how living organisms and their intricate parts could have been created. Unlike the intelligent human designers of artificial things, there is no godlike intelligent designer of living things, just the sustained operation of natural selection:

. . . the blind, unconscious, automatic process which Darwin discovered, and which we now know is the explanation for the existence and apparently purposeful form of all life. If it can be said to play the role of watchmaker in nature, it is the *blind* watchmaker. (Dawkins, 1987, p. 5)

In his more recent book, *Flights of Fancy: Defying Gravity by Design and Evolution*, Dawkins focuses on the origin of flight—by animals and airplanes. Here too, he asserts that radically different “research and development” processes are involved in their provenance:

Animals have become flying machines through millions of years of slow, gradual improvement, getting better at it as the generations went by. Humans have built better and better flying machines through successive designs on drawing boards, and the improvements have taken place on a timescale of years and decades, instead of millions of years. (Dawkins, 2021, p. 251)

Nevertheless, beyond this stark contrast, Dawkins (2021) does fleetingly entertain an intriguing parallel: “Perhaps engineers’ new ideas . . . are rather like mutations. These ‘idea mutations’ are subject to something like natural selection” (p. 254), in which the unsuccessful ideas will be discarded, and the successful ideas will proceed to production. Readers acquainted with the writings of philosopher Karl Popper will find this notion familiar. Popper (1978), one of the 20th century’s most influential philosophers of science, maintained that our *ideas* are more expendable than *ourselves*: “Let our conjectures, our theories, die in our stead” (p. 354). Like Dawkins, Popper too aligned this idea with natural selection. We would, of course, more appropriately associate this idea with selection by reinforcement (see Dennett, 1975). Unfortunately, Dawkins never seriously pursues this promising possibility.

But Dawkins does move on to another key issue—one that involves the role of cultural selection in human innovation. He notes the alluring myth of the “lone genius” persistently pursuing a fresh idea or product until it reaches its final form (see Berkun, 2007, for more on this and other myths of innovation). Nonetheless, he recognizes that this rather dreamy account misses another important way that innovations evolve. Take the case of humans’ fabrication of flying machines, for example: “Planes evolved gradually, with roots in gliders and continuing on through early biplanes to the sleek, fast, graceful airliners of today” (Dawkins, 2021, p. 263). Numerous individuals across many generations boosted this evolutionary process. No single person can properly claim ownership in instances like this (also see Wasserman & Scerri, 2017).

### Dennett’s Speculations

Let’s now consider one more eminent evolutionary thinker who even more thoroughly discusses the importance of cultural selection than Dawkins. In his book, *From Bacteria to Bach and Back: The Evolution of Minds*, philosopher Daniel Dennett underscores the pivotal role cultural evolution plays in enabling humans to craft magnificent creations, for example, Antoni Gaudí’s *Sagrada Familia*. For Dennett, the key to this creative process is language: “a wonderful medium for manipulating others, and manipulating yourself. And as the products of the cultural evolutionary process have accumulated, they have enabled us to become *truly intelligent designers*, with forethought, planning and so on” (Dennett, 2017, p. 42, emphasis added).

Dennett invites us to compare Gaudí’s basilica in Barcelona with a termite colony, an intricate structure that uncannily resembles the *Sagrada Familia*. “They look similar, but Gaudí’s church is a product of intelligent design; it’s top-down, with a charismatic boss who thought it out in advance. There is no Gaudí in the termite castle” (Dennett, 2017, p. 42).

Alas, Dennett’s argument takes us right back to Darwin and Dawkins: denying an omniscient divine designer for natural things, but accepting a prospicient human designer for artificial things. Not only that, but Dennett’s formulation also presents us with a new puzzle: we must now recognize that the brain “between our ears is more like a termite colony than you might be happy thinking. The latest count is 86 billion neurons, each more clueless than a termite, with no boss” (Dennett, 2017, p. 42). How might one imagine organizing those 86 billion neurons into a human mind?

### Rosenbaum’s Proposal

It just so happens that cognitive psychologist David Rosenbaum has proposed a stunning solution to Dennett’s puzzle—one that’s thematically compatible with selectionist thinking. In his 2014 book, *It’s a Jungle in There: How Competition and Cooperation in the Brain Shape the Mind*, Rosenbaum asserts that Darwin’s evolutionary theory should be extended to psychology. Of course, the major challenge to this extension is explaining intelligent action without invoking an intelligent designer.

To do so, Rosenbaum unceremoniously gives Dennett’s brainy “boss” the boot. In Rosenbaum’s account, the population of neurons and neural systems (metaphorical “beings”) in the brain needs no executive at all to promote adaptive action. Think *E pluribus unum* (Out of many one): “This view of yourself as a population made up of beings, none as intelligent as you but collectively comprising you with no one in charge, is the view I wish to advance” (Rosenbaum, 2014, p. 10). According to Rosenbaum, the fittest deeds and ideas will arise solely from these neural systems competing and cooperating with one another—his *Jungle Principle*.

### Returning to Skinner

The various cognitive and neural theoretics of Dawkins, Popper, Dennett, and Rosenbaum do seem to be antithetical to Skinner’s own behavior theory, because they in one way or another appeal to unobservable events. Yet, in a surprising and controversial theoretical extension (Baum, 2011; Catania, 2011), Skinner himself applied the principles of reinforcement to the world *within the skin:*

An adequate science of behavior must consider events taking place within the skin of the organism, not as physiological mediators of behavior, but as part of behavior itself. It can deal with these events without assuming that they have any special nature or must be known in any special way. The skin is not that important as a boundary*.* Private and public events have the same kinds of physical dimensions. (Skinner, 1969, p. 228)

Of course, various aspects of the cognitive, neural, and behavioral accounts advanced by Dawkins, Popper, Dennett, Rosenbaum, and Skinner to explain human creativity and innovation are highly speculative and may strikingly conflict with one another. Nonetheless, if any one of these or still other selectionist perspectives is to be adopted in psychology (see Hanisch et al., 2025), then Rosenbaum (2014) wisely counsels that, “we must accept the ruthlessness of the scrutiny to which it should be subjected, for that is the Darwinian way. It is the nature of science to take this hard-headed approach” (p. 194).

With Rosenbaum’s counsel in mind, I firmly believe that Skinner’s tripartite theory of selection by consequences represents the most comprehensive and compelling of current accounts for explaining human creativity and innovation. Decoupling purposeful and foresightful design from natural selection and selection by reinforcement as well as affording proper prominence to cultural selection should dramatically advance our grasp of evolution at all levels: phylogenic, ontogenic, and sociogenic. It should also free us from the mentalism that so commonly creeps into our theories of human creativity and innovation (Cofnas, 2024).

## Mentalism and Selectionism

It is by now obvious that mentalism has permeated discussions of creativity and innovation since Paley. The monumental efforts of Darwin and Skinner to decouple design from selection have not yet succeeded. Why? Didn’t Huxley (1942) declare the matter to have been settled over 8 decades ago?

It was one of the great merits of Darwin himself to show that the purposiveness of organic structure and function was apparent only. The teleology of adaptation is a pseudo-teleology, capable of being accounted for on good mechanistic principles, without the intervention of purpose, conscious or subconscious, either on the part of the organism or of any outside power. (p. 412)

Nevertheless, when the time came for Darwin to name the process that shapes organisms via the vicissitudes of survival, the phrase “natural selection” may have been a most infelicitous choice. Yes, natural selection was easier for Darwin to communicate to the naturalist public than other expressions he considered; but it may have opened the door to persistent misconception.

His one-time rival turned comrade-in-arms, Alfred Russell Wallace, was particularly critical. In a letter to Darwin, sent after publication of the *Origin*, Wallace argued—no doubt with a wink—that the metaphor was not well “adapted” to convey his theory of evolution to the public. He was concerned that the word “selection” encouraged readers to view nature as a forward-looking, intelligent designer that was shaping the evolutionary course of life. (Cooperrider, 2016)

Wallace’s lengthy letter of July 2, 1866, carefully explained that, to those who were intimately acquainted with the details of Darwin’s theory, the meaning of natural selection was “as clear as daylight” and “beautifully suggestive.” However, to others who were less well acquainted with his theory, the term was a serious “stumbling block.” Wallace was especially concerned that Darwin’s term could be viewed as “personifying” nature as well as that “natural selection” itself was misleading, because nature “does not so much *select* special variations, as *exterminate* the most unfavourable ones.”

In its place, Wallace proposed using Herbert Spencer’s earlier and more graphic term “survival of the fittest.” This phrase represents the “plain expression of the facts” and “could not possibly be denied or misunderstood,” unlike natural selection, which Wallace insisted already “*is* misunderstood & apparently always will be.”

Darwin appreciated Wallace’s criticisms and addressed them in his 1868 book, *The Variation of Animals and Plants Under Domestication.* “The term ‘natural selection’ is in some respects a bad one, as it seems to imply conscious choice; but this will be disregarded after a little familiarity” (p. 6). Would that it were so!

I raise this serious terminological matter not to solve it, but rather for today’s readers to recognize that we are engaged in a most consequential debate between conflicting ideas, in which the central concept under consideration—selection—has from the outset had unwelcome mentalistic connotations.

### Coda

Circling back to the opening quotations of Dennett and Donahoe, permit me to leave you with the following pointed claims: our own minds cannot be exempt from an evolutionary explanation. Furthermore, this explanation need not invoke such mentalistic notions as design and intention. Selection by consequences may suffice.

## Data Availability

No data are reported.
